# Kainic Acid Induces mTORC1-Dependent Expression of *Elmo1* in Hippocampal Neurons

**DOI:** 10.1007/s12035-016-9821-6

**Published:** 2016-03-19

**Authors:** Magdalena Blazejczyk, Matylda Macias, Michal Korostynski, Marcelina Firkowska, Marcin Piechota, Agnieszka Skalecka, Aleksandra Tempes, Alicja Koscielny, Malgorzata Urbanska, Ryszard Przewlocki, Jacek Jaworski

**Affiliations:** 1 International Institute of Molecular and Cell Biology, 4 Ks. Trojdena St., 02-109 Warsaw, Poland; 2 Institute of Pharmacology, Polish Academy of Sciences, 12 Smetna St, 31-343 Krakow, Poland

**Keywords:** Epilepsy, Rapamycin, Gene expression, mTORC1, Elmo1

## Abstract

Epileptogenesis is a process triggered by initial environmental or genetic factors that result in epilepsy and may continue during disease progression. Important parts of this process include changes in transcriptome and the pathological rewiring of neuronal circuits that involves changes in neuronal morphology. Mammalian/mechanistic target of rapamycin (mTOR) is upregulated by proconvulsive drugs, e.g., kainic acid, and is needed for progression of epileptogenesis, but molecular aspects of its contribution are not fully understood. Since mTOR can modulate transcription, we tested if rapamycin, an mTOR complex 1 inhibitor, affects kainic acid-evoked transcriptome changes. Using microarray technology, we showed that rapamycin inhibits the kainic acid-induced expression of multiple functionally heterogeneous genes. We further focused on engulfment and cell motility 1 (Elmo1), which is a modulator of actin dynamics and therefore could contribute to pathological rewiring of neuronal circuits during epileptogenesis. We showed that prolonged overexpression of *Elmo1* in cultured hippocampal neurons increased axonal growth, decreased dendritic spine density, and affected their shape. In conclusion, data presented herein show that increased mTORC1 activity in response to kainic acid has no global effect on gene expression. Instead, our findings suggest that mTORC1 inhibition may affect development of epilepsy, by modulating expression of specific subset of genes, including *Elmo1*, and point to a potential role for Elmo1 in morphological changes that accompany epileptogenesis.

**Electronic supplementary material:**

The online version of this article (doi:10.1007/s12035-016-9821-6) contains supplementary material, which is available to authorized users.

## Introduction

Epilepsy is a common chronic neurological disorder that is characterized by recurrent seizures that are unpredictable and sometimes progressively severe [1]. Epilepsy is triggered by various environmental and genetic factors [2, 3] that induce numerous molecular changes, including changes in gene expression, that drive epileptogenesis [4, 5]. An important part of epileptogenesis is the pathological rewiring of neuronal circuits that involves changes in neuronal morphology (e.g., dendritic spine rearrangement and axonal sprouting; [6, 7]).

Several lines of evidence suggest links between epileptogenesis and complex 1 of mammalian/mechanistic target of rapamycin (mTOR), a serine-threonine protein kinase [8, 9]. On one hand, status epilepticus induces mTOR activation [10–13]. On the other hand, rapamycin, an mTOR inhibitor, has antiepileptogenic effects in kainic acid (KA)-induced and pilocarpine-induced models of epilepsy in animals [13–15]. Among other cellular roles, mTOR complex 1 (mTORC1) is known to control the transcription of several genes [16]. Our previous studies found increased nuclear levels of phosphorylated mTOR in hippocampal and cortical neurons cultured in vitro and in the brain in vivo after application of KA [11]. This observation could suggest that mTOR contributes to epileptogenesis by regulating gene expression. To test this hypothesis, in the present study, we aimed to evaluate effects of rapamycin on KA-evoked transcriptome changes in organotypic hippocampal slices using a microarray technology. The analysis revealed that mTORC1 is not a major driving force behind transcriptome changes induced by KA. Nevertheless, we found effects of rapamycin on the KA-induced expression of multiple functionally heterogeneous genes. One of them was *engulfment and cell motility 1* (*Elmo1*), which duration of KA-induced expression was inhibited by rapamycin.

Elmo1, in complex with RhoG and Dock180, induces the activation of small GTPase Rac1 [17], a major regulator of actin dynamics [18]. Reorganized F-actin networks have been postulated to lead to the long-term stabilization of synaptic changes and consolidation of enhanced neuronal activity. Thus, alterations in F-actin network may be related to the aberrant hyperexcitability that is observed in epilepsy [19]. Rac1 and RhoA were significantly upregulated in the pentylenetetrazole kindling model and play a role in aberrant mossy fiber sprouting [20]. Links between Rac and epilepsy were further revealed by the impairment of hippocampal circuitry and epileptic phenotype of Rac1/Rac3 double-knockout mice [21]. Nonetheless, however, the role of Rho-family small GTPases and actin reorganization in epilepsy is poorly understood and the role of Elmo1 in epilepsy has not yet been elucidated. Thus, in the second part of this study, we investigated in more details effects of prolonged overexpression of *Elmo1* in cultured hippocampal neurons and showed increased axonal growth, decreased dendritic spine density, and affected their shape.

## Materials and Methods

### Antibodies and Reagents

The antibodies that were used in the study are listed in Table 1. The following reagents were used for pharmacological treatments: dimethylsulfoxide (DMSO; Sigma-Aldrich, St. Louis, MO, USA), rapamycin (R; LC Laboratories, Woburn, MA, USA), and kainic acid (KA, Tocris Bioscience, Ellisville, MO, USA). Hoechst 33342 was purchased from Life Technologies (Carlsbad, CA, USA).

**Table 1:** Antibodies used in the study

Primary antibody	Host	Manufacturer; catalog no.; dilution	Secondary antibody
Enhanced chemiluminescent (ECL) detection			
Anti-phospho-S6 (P-S6) Ser235/236	Rabbit	Cell Signaling Technology, Beverly, MA; #4858; 1:500 in 5 % BSA in TBS-T^a^	Anti-rabbit IgG, HRP-conjugated; Cell Signaling Technology, Beverly, MA; #7074; 1:10000 in 5 % nonfat milk in TBS-T
Anti-S6 (S6)	Rabbit	Cell Signaling Technology, Beverly, MA; #2217; 1:1000 in 5 % BSA in TBS-T	
Anti-mTOR	Rabbit	Cell Signaling Technology, Beverly, MA; #2983; 1:1000 in 5 % BSA in TBS-T	
Anti-caspase-3	Rabbit	Cell Signaling Technology, Beverly, MA; #9662; 1:1000 in 5 % BSA in TBS-T	
Anti-GFP	Rabbit	MBL International Corporation, Geel, Belgium; #598; 1:2000 in 5 % nonfat dry milk in TBS-T	
Anti-Elmo1	Rabbit	Eurogentec, Liege, Belgium; custom order^b^; 1:500 in 5 % nonfat dry milk in TBS-T	
Anti-α-tubulin	Mouse	Sigma-Aldrich, St. Louis, MO; #T5168; 1:20,000 in 5 % nonfat dry milk in TBS-T	Anti-mouse IgG, HRP-conjugated; Cell Signaling Technology, Beverly, MA; #7076; 1:10000 in 5 % nonfat milk in TBS-T
Anti-β-actin-HRP	Mouse	Sigma-Aldrich, St. Louis, MO; A3854; 1:15,000 in 5 % nonfat dry milk in TBS-T	N/A
Immunofluorescence			
Anti-phospho-S6 (P-S6) Ser235/236	Rabbit	Cell Signaling Technology, Beverly, MA; #4858; 1:200 in 1 % donkey serum in PBS-T^c^	Anti-rabbit IgG Alexa Fluor 488; Life Technologies, Carlsbad, CA; A-21206; 1:500 in 1 % donkey serum in PBS-T
Anti-c-Fos	Rabbit	Santa Cruz Biotechnology, Santa Cruz, CA; #7202; 1:200 in 1 % donkey serum in PBS-T	
Anti-GFAP	Mouse	Millipore, Darmstadt, Germany; #04-1031; 1:500 in 1 % donkey serum in PBS-T	
Anti-GFP	Mouse	MBL International Corporation, Geel, Belgium; #598; 1:1000 in GDB buffer^d^	

^a^Tris-buffered saline (TBS-T) (50 mM Tris, 150 mM NaCl), pH 7.6 + 0.1 % Tween 20

^b^Elmo1 peptide sequence taken for rabbit immunization: aa 109–124: GDLEESPQGEVPHDSL

^c^Phosphate-buffered saline (PBS-T) (137 mM NaCl, 2.7 mM KCl, 10 mM Na_2_HPO_4_, 2 mM KH_2_PO_4_), pH 7.4 + 0.2 % Triton X-100

^d^GDB buffer (0.2 % gelatin, 0.8 M NaCl, 0.5 % Triton X-100, 30 mM phosphate buffer), pH 7.4)

### DNA Constructs

The mammalian expression plasmids β-actin-green fluorescent protein (GFP) and pSuper have been described previously [22, 23]. β-Actin-tdTomato plasmid, which encodes tdTomato under control of the β-actin promoter, was obtained by subcloning the tdTomato coding sequence using EcoRI/BamHI [24] to modified β-actin-16pl [25]. β-Actin-Elmo1-GFP was obtained by subcloning the rat *Elmo1* coding sequence to EcoRI/SalI sites of β-actin-GFP. The coding sequence of *Elmo1* was obtained from rat neuronal complementary DNA (cDNA) by polymerase chain reaction (PCR) using the following primers: 5′-GAGAATTCGTAATGCAGGTGGTGAAG-3′ and 5′-TGGTCGACTCAGTTACAGTCATAAACA-3′. pSuper-sh#Elmo1 was obtained by subcloning the previously described *Elmo1* short hairpin RNA (shRNA) coding sequence [26] to pSuper.

### Organotypic Hippocampal Slices

**Fig. 1:**
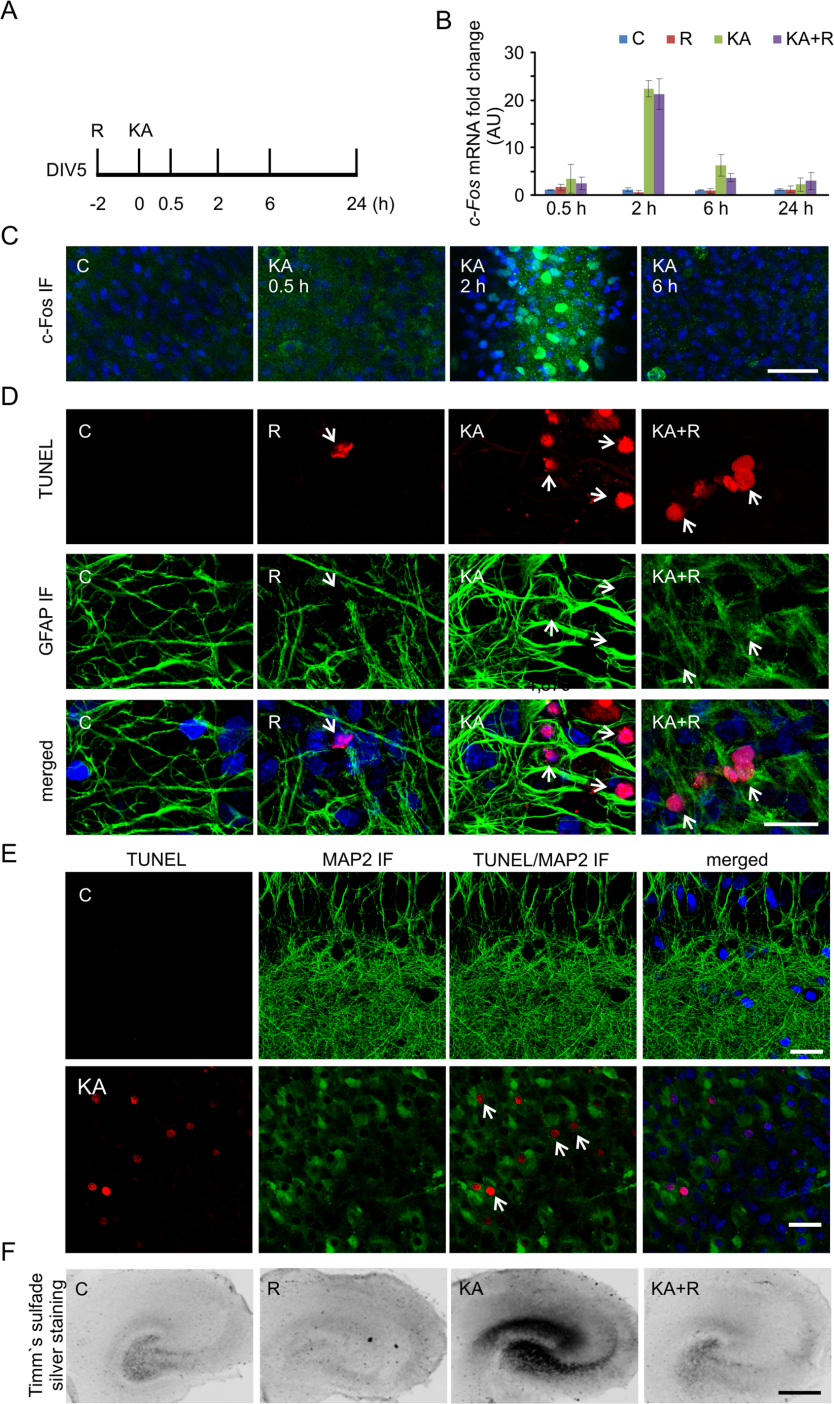
In vitro model system to study molecular and cellular responses to kainic acid. **a** Scheme of rat organotypic hippocampal slice treatments with kainic acid (KA) and rapamycin (R). **b** Expression of *c-Fos* mRNA after KA treatment analyzed by qRT-PCR. *C* control, *R* rapamycin, *KA* kainic acid, *KA*+*R* kainic acid+rapamycin. Number of cultures (*N*) = 3, number of slices per condition per culture = 4. **c** Representative confocal images of CA1/CA3 area of organotypic hippocampal slices immunofluorescently stained for c-Fos (*green*) after KA at the indicated time points. Nuclei were counterstained with Hoechst 33342 (*blue*). *Scale bar* = 50 μm. **d** Representative confocal images of CA1/CA3 area of organotypic hippocampal slice TUNEL staining (*red*) 24 h after indicated treatment. Slices were additionally immunofluorescently stained with the glial marker GFAP (*green*) and counterstained with nuclear dye Hoechst 33342 (*blue*). *Scale bar* = 20 μm. **e** Representative confocal images of CA1/CA3 area of organotypic hippocampal slice TUNEL staining (*red*) 24 h after indicated treatment. Slices were additionally immunofluorescently stained with the neuronal marker MAP2 (*green*) and counterstained with nuclear dye Hoechst 33342 (*blue*). *Scale bar* = 20 μm. **f** Representative images of organotypic hippocampal slice with Timm’s staining treated as indicated. *Scale bar* = 500 μm (color figure online)

Hippocampal slice cultures were prepared based on the method described by Stoppini et al. [27] and in conformity with institutional guidelines of the First Local Ethics Committee in Warsaw (decision no. 861/2007), which are in compliance with the European Community Council Directive (86/609/EEC). Briefly, 8- to 10-day-old Wistar rat pups were decapitated without anesthesia and the hippocampi were dissected. Sections (400 μm) were prepared using a McIlwain tissue chopper (Ted Pella, Redding, CA, USA) and separated under ice-cold Gey’s balanced salt solution (Sigma-Aldrich; supplemented with 5 mg/ml glucose and 1 % penicillin/streptomycin). Slices were plated on semiporous membranes (four per membrane; Millipore, Darmstadt, Germany) and maintained at 37 °C in 5 % CO_2_ in Minimum Essential Medium (MEM) supplemented with Earle’s salts (MP Biomedicals, Santa Ana, CA, USA) and 25 % Hanks’ balanced salt solution (HBSS; MP Biomedicals), 25 % heat-inactivated horse serum (Sigma-Aldrich), 5 mg/ml glucose, 1 mM glutamine, and 1 % penicillin/streptomycin (all from Sigma-Aldrich). The maintenance media was changed every 3–4 days. For pharmacological treatments, slices on day 5 in vitro (DIV5) were treated either with vehicle (DMSO) or 10 μM KA (Fig. 1a). To inhibit mTOR activity, 20 nM rapamycin was added to slices either alone or in combination with KA (Fig. 1a). Rapamycin was added 2 h prior to KA. Slices were collected 0.5, 2, 6, and 24 h after KA treatment (Fig. 1a). For the analysis of aberrant axonal sprouting, DIV10 slices were treated with 6 μM KA for 48 h. Rapamycin was added 2 h prior to KA and present during KA treatment. The medium was then changed to maintenance medium, and slices were cultured until DIV26.

### Animals and Pharmacological Treatment

For the pharmacological experiments, 3-month-old male Wistar rats were used and described procedures were conducted in conformity with the institutional guidelines of the First Local Ethics Committee in Warsaw (decision no. 76/2010), which are in compliance with the European Community Council Directive (86/609/EEC). Tissue for messenger RNA (mRNA) analysis was collected during experiments described by Macias et al. [11]. Briefly, the animals were divided into four groups: vehicle, KA, rapamycin, and rapamycin+KA. Rapamycin was initially dissolved in 100 % ethanol for long-term storage. Prior to the injection, rapamycin was diluted in a vehicle solution that contained 5 % Tween 80 and 5 % PEG 400 (low-molecular-weight grade of polyethylene glycol; Sigma) and injected intraperitoneally (i.p.; 10 mg/kg) three times per week for 4 weeks. A control group of rats was injected with vehicle solution (5 % Tween 80, 5 % PEG 400, and 4 % ethanol). KA (10 mg/kg) was delivered by i.p. injection. In the rapamycin+KA group, KA was injected 48 h after the last dose of rapamycin. KA-injected animals were observed, and their behavior was digitally recorded. Severity of seizures was assessed according to a modified Racine’s scale [28]. Only the animals that displayed three episodes of generalized seizures (stage 3) were further analyzed. The rats were sacrificed 2 h following KA injection. Next, cortices and hippocampi were dissected from the brains and stored in RNAlater (Life Technology, Carlsbad, CA, USA) in −80 °C until use.

### Primary Hippocampal and Cortical Neuron Cultures, Transfection, and Pharmacological Treatment

Animals that were used to obtain neurons for cell culture were sacrificed according to the protocol approved by the First Ethical Committee in Warsaw, Poland (decision no. 76/2010). Primary hippocampal and cortical cultures were prepared from embryonic day 19 (E19) rat brains as described previously [29]. Cells were plated on coverslips coated with poly-L-lysine (37.5 μg/ml, Sigma-Aldrich) and laminin (2.5 μg/ml, Roche, Basel, Switzerland) at a density of 500 cells/mm^2^ (hippocampal) or precoated BioCoat plastic plates (BD Biosciences Discovery Labware, San Jose, CA, USA) at a density of 1250/mm^2^ (cortical). Neuronal cultures were grown in Neurobasal medium (Life Technologies) supplemented with B27 (Life Technologies), 0.5 mM glutamine, 12.5 μM glutamate, and penicillin/streptomycin mix (all from Sigma-Aldrich). For the morphology experiments on day in vitro (DIV) 1, 8, or 18 hippocampal cultures were transfected using Lipofectamine2000 (Life Technologies) for 3 days as described previously [30]. In the overexpression experiments, β-actin-GFP or β-actin-Elmo1-GFP was cotransfected with β-actin-tdTomato at a 3:1 ratio. To visualize dendritic protrusions, the cells were stained with GFP tags. To characterize anti-Elmo1 antibody, cortical cultures were transfected on DIV0 using an Amaxa Nucleofector II Device and Amaxa Rat Neuron Nucleofactor Kit (Lonza, Basel, Switzerland) according to a modification of the manufacturer’s protocol [31] with pSuper or pSuper-sh#Elmo1 and β-actin-GFP or β-actin-Elmo1-GFP. Four days later, the cells were lysed.

For pharmacological treatments on DIV14, cortical cultures were treated with either vehicle (DMSO, control) or 25 μM KA for 10 min. To inhibit mTOR activity, 20 nM rapamycin was added to the cultures either alone or in combination with KA. Cortical cultures were collected 4 and 24 h after KA treatment and then lysed in sodium dodecyl sulfate (SDS) sample buffer for Western blot analysis or RNAlater for reverse-transcription quantitative real-time polymerase chain reaction (qRT-PCR) analysis.

### Immunofluorescent Labeling

For the immunofluorescent staining of overexpressed proteins, neurons were fixed with 4 % paraformaldehyde (PFA) that contained 4 % sucrose in phosphate-buffered saline (PBS) for 10 min at room temperature. After fixation, the cells were washed three times with PBS for 5 min at room temperature and incubated with primary antibodies in GDB buffer (0.2 % gelatin, 0.8 M NaCl, 0.5 % Triton X-100, and 30 mM phosphate buffer, pH 7.4) overnight at 4 °C. The cells were then washed three times with PBS for 10 min at room temperature. Secondary antibodies were applied in GDB for 1 h at room temperature and underwent three washes with PBS, 10 min each. Secondary antibodies that were conjugated to Alexa Fluor 488 were used for labeling.

For immunofluorescent staining, organotypic slices were fixed with 4 % PFA that contained 4 % sucrose in PBS for 24 h at 4 °C. The slices were then washed with PBS that contained 0.2 % Triton-X100 (PBS-T), blocked with 5 % normal donkey serum in PBS-T, and incubated overnight at 4 °C with antibodies described in Table 1, diluted in 1 % normal donkey serum (Sigma-Aldrich) in PBS-T. Afterward, the slices were rinsed with PBS-T and labeled for 1 h at room temperature with the respective secondary antibodies conjugated to Alexa Fluor dyes diluted 1:500 in 1 % donkey normal serum in PBS-T. After several washes with PBS-T, the slices were mounted on glass slides, air-dried, and sealed with Vectashield with Hoechst 33342 Mounting Medium (Vector Laboratories, Peterborough, UK). Results of c-Fos IF (Fig. 1c) were obtained from two independent slice cultures (eight animals). Slices from each animal were exposed to all types of treatment. At least two slices per animal per condition were analyzed. Results of P-S6 IF (Fig. 1d) were obtained from two independent slice cultures (three slices/condition/preparation were analyzed).

### Terminal Deoxynucleotidyl Transferase dUTP Nick End Labeling Assay

The terminal deoxynucleotidyl transferase dUTP nick end labeling (TUNEL) assay was performed using the In Situ Cell Death Detection Kit, TMR red (Roche Applied Science, Mannheim, Germany). Organotypic hippocampal slices were permeabilized for 2 min on ice with permeabilization solution (0.1 % Triton X-100 and 1 % sodium citrate) and incubated with labeling solution plus enzyme solution at 37 °C for 1 h. As a negative control, the enzyme solution was omitted. The slices were then washed three times with PBS-T and counterstained with Hoechst 33342 dye and anti-GFAP antibody as described above. TUNEL staining was performed on two independent slice cultures (eight animals). Slices from each animal were exposed to all types of treatment. At least two slices per animal per condition were analyzed.

### Confocal Imaging and Analysis

Images of immunofluorescently or TUNEL-stained neurons were acquired using confocal microscopy. Confocal images were obtained with sequential acquisition settings at 1024 × 1024 pixel resolution using a Zeiss LSM 5 Exciter microscope equipped with Ar (488 and 514 nm), HeNe (543 and 633 nm), and diode (405 nm) lasers. The laser power and image acquisition settings were kept constant. Each image was a z-series of images, each averaged twice. The obtained stack was “flattened” into a single image using maximum projection.

Morphometric analysis of axon and dendrite length, the number ends of axons and dendrites, and density, width and length of dendritic protrusions (spines and filopodia) of neurons in dissociated in vitro cultures was performed using MetaMorph image analysis software (Universal Imaging Corporation, Downingtown, PA, USA). For the total axon length and axonal ends, the full axonal tree of each neuron was traced on images acquired at a ×10 objective (0.6 digital zoom). The number of dendrites and dendrite length were quantified on images that were acquired with a ×40 objective (0.7 digital zoom) as described previously [23]. Quantification of the density, width, and length of dendritic protrusions was performed on images acquired with a ×40 objective (×5 digital zoom). The density of protrusions was counted as the number of protrusions on the 10 μm dendrite and three dendritic segments of one neuron. The average length, protrusion width, and density per cell for each cell were calculated and used for further averaging. Twenty randomly chosen cells were examined for each experimental condition for each culture batch. Data were obtained from three (analysis of axons or dendrites) or two (analysis of dendritic protrusions) independent batches of neurons. For further classification of protrusions, the protrusions were divided arbitrarily into two classes: filopodia (head width ≤ 0.4 μm) and spines (head width > 0.4 μm). The densities of filopodia and spines were next calculated as their number on the 10 μm of dendrite and expressed as percentage of all counted protrusions. Comparison of the protrusions length/width ratio were done as described by Cymerman et al. [32], and data are presented as % cumulative change.

### Tissue Collection, RNA Preparation, and Gene Expression Profiling

To isolate RNA from organotypic hippocampal slices, at the end of the experiments, slices were placed in RNAlater reagent (Life Technology) and stored at −80 °C until use. RNA was isolated using the RNeasy Mini Kit (Qiagen, Venlo, The Netherlands) according to the manufacturer’s protocol. The total RNA concentration was measured using a NanoDrop Spectrometer 2000 (Thermo Fisher Scientific, Waltham, MA, USA), and its quality was determined by chip-based capillary electrophoresis using an RNA 6000 Nano LabChip Kit and Agilent Bioanalyzer 2100 (Agilent, Palo Alto, CA, USA) according to the manufacturer’s instructions. RNA from three independent biological repeats was pooled to create a sample for each microarray (e.g., single sample from one biological repeat that consisted of eight slices, each from different newborn rats of the same litter). A starting amount of 800 ng high-quality (RNA integrity numbers 9.8–9.9) total RNA (equally pooled from three independent experiments) was used to generate cDNA and complementary RNA (cRNA) with the Illumina TotalPrep RNA Amplification Kit (Illumina, San Diego, CA, USA). The obtained cDNA served as a template for in vitro transcription with T7 RNA polymerase and biotin UTP to generate multiple copies of biotinylated cRNA. Each cRNA sample (1.5 μg) was hybridized overnight to a RatRef-12 BeadChip array (Illumina). The chips were then washed, dried, and scanned with the BeadArray Reader (Illumina). Three biological replicates were used per time point and 12 arrays per each treatment group; the number of control samples was doubled. The treatment group samples were equally distributed between array plates and hybridization batches. Raw microarray data were generated using BeadStudio 3.0 (Illumina).

### Microarray Data Analysis

Microarray quality control was performed using the BeadArray R package. The following parameters were checked on all 60 arrays: number of outliers, number of beads, and percentage of detected probes. After background subtraction, the data were normalized using quantile normalization and then log2-transformed. The obtained signal was taken as the measure of mRNA abundance derived from the level of gene expression. Statistical analysis of the results was performed using two-way analysis of variance (ANOVA), with KA and time as factors, followed by Benjamini and Hochberg correction for multiple testing (percent FDR; [33]). The effects of rapamycin on KA-induced alterations in gene expression are presented as differences in fold changes. All of the statistical analyses were performed using R software [34]. Hierarchical clustering was performed with dChip software using the Euclidean distance and average linkage method. The functional annotation analysis tool DAVID 2008 was used to identify overrepresented ontologic groups among the gene expression patterns and group genes into functional classifications [35]. The identification of overrepresented transcription factor binding sites (TFBSs) was performed using the cREMaG database with default parameters [36].

### Reverse-Transcription Quantitative Real-Time Polymerase Chain Reaction

RNA from organotypic hippocampal slices, hippocampi or cultured neurons was isolated with the RNeasy Protect Mini Kit (Qiagen). cDNA was prepared with High Capacity RNA-to-cDNA Master Mix (Life Technologies) according to the manufacturer’s protocol. qRT-PCR was performed with a 7900HT machine and TaqMan Array Fast Plates (Life Technologies) with the following TaqMan rat probes: *Tubb6* (Rn01253666_m1), *Bdnf* (Rn01484924_m1), *Elmo1* (Rn01528402_m1), *Lrrfip2* (Rn01245379_m1), *Npas4* (Rn00596522_m1), *Nr4a3* (Rn00581189_m1), *Abra* (Rn00598518_m1), *Adrb1* (Rn00824536_s1), *Cdr2* (Rn01511457_m1), *Vgf* (Rn00578591_g1), *Gprc5a* (Rn01459296_m1), *Gadd45g* (Rn01435432_g1), *Nfil3* (Rn01434874_s1), *Scg2* (Rn01400686_g1), *Egr4* (Rn00569509_g1), and *c-Fos* (Rn02396759_m1). *Actb* (Rn00667869_m1) or *Gapdh* (Rn99999916_s1) was used as an internal control. SDS 2.4 and RQ Manager 1.2.1 programs were used for data acquisition and preliminary analysis.

### Preparation of Protein Extracts and Western Blot Analysis

**Fig. 2:**
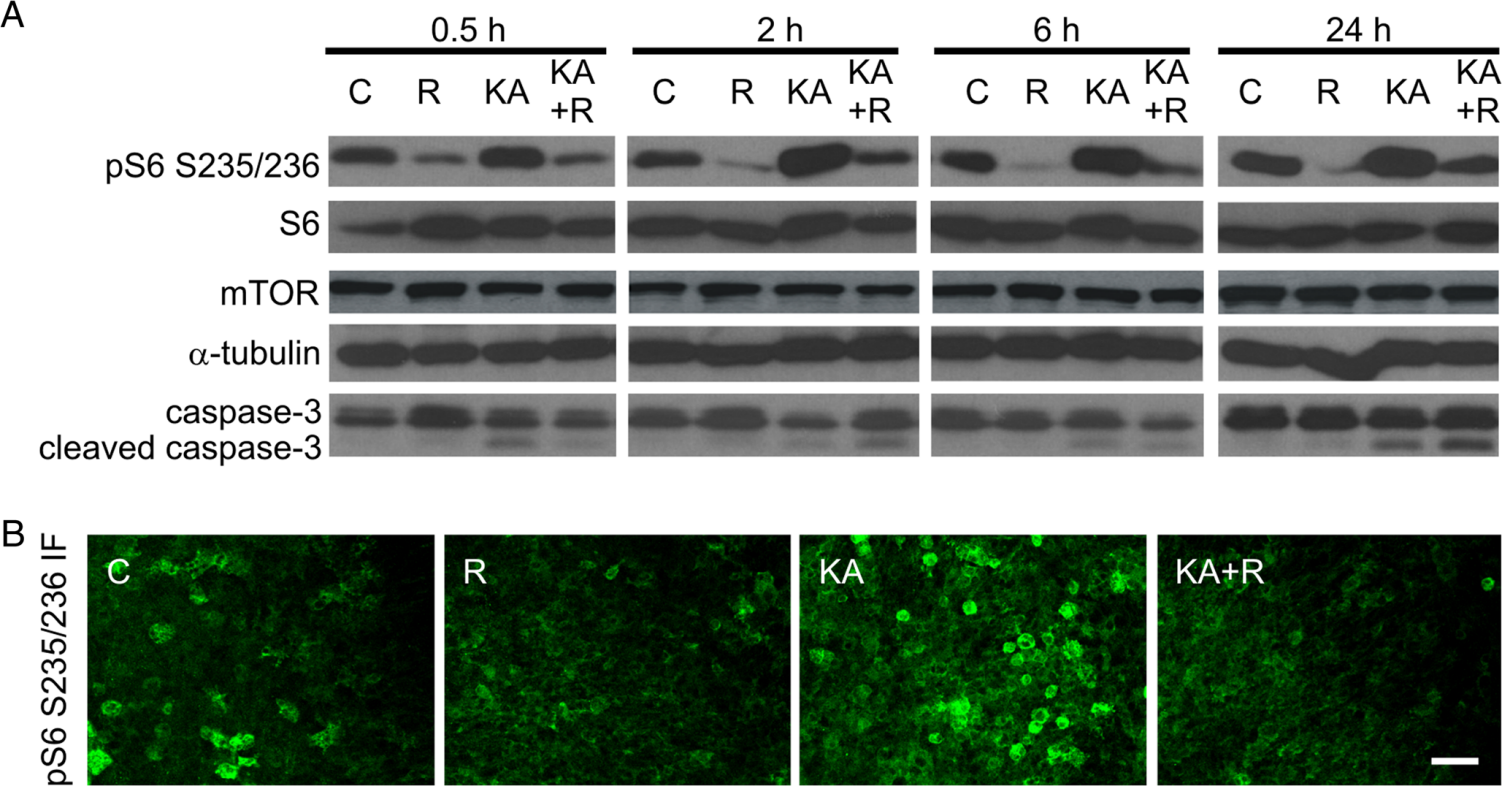
Kainic acid induces mTOR activity and cell death in organotypic hippocampal slices. **a** Western blot analysis of phosphorylated ribosomal protein S6 (P-S6; Ser235/236), mTOR, caspase-3, and cleaved caspase-3 in protein lysates obtained from organotypic slices treated as indicated. α-Tubulin is shown as a loading control. *C* control, *R* rapamycin, *KA* kainic acid, *KA*+*R* kainic acid+rapamycin. **b** Representative confocal images of CA1/CA3 area of organotypic hippocampal slice immunofluorescently stained for P-S6 2 h after R, KA, or KA+R treatment. *Scale bar* = 50 μm

For the biochemical studies, organotypic hippocampal slices or cortical cultures were collected and stored at −80 °C until use. Cortical cultures were lysed in SDS sample buffer. To extract proteins from slices, the slices were lysed in homogenization buffer (10 mM Tris, pH 7.6, 150 mM NaCl, 2 mM EDTA, and 0.3 % 3-[(3-cholamidopropyl)dimethylammonio]-1-propanesulfonate [CHAPS]) supplemented with protease (Roche, Indianapolis, IN, USA) and phosphatase inhibitors (Sigma-Aldrich) and centrifuged at 14,000×*g* for 30 min at 4 °C. The supernatants were collected, and the protein concentration in the obtained protein lysates was measured using the DC Protein Assay (Bio-Rad Laboratories, Hercules, CA, USA). The proteins from both types of experiments were then analyzed by Western blot. After protein electrotransfer, the membranes were blocked for 1 h at room temperature in 5 % nonfat dry milk in TBS-T (Tris-buffer saline, pH 7.6, with 0.1 % Tween 20) and incubated overnight at 4 °C with primary antibodies (see Table 1). For enhanced chemiluminescent detection (ECL), the next day, the membranes were washed several times with TBS-T and incubated for 1 h with horseradish peroxidase (HRP)-conjugated secondary antibodies diluted in TBS-T that contained 5 % nonfat dry milk (see Table 1). Finally, the membranes were washed with TBS-T, incubated for 1 min with ECL reagent, and immediately exposed to X-ray film (Sigma-Aldrich). Western blot analysis of levels of phosphorylated ribosomal protein S6 (P-S6; Ser235/236), mTOR, caspase-3, and cleaved caspase-3 in organotypic slices (Fig. 2a) was performed on protein lysates obtained from three independent slice culture preparations (eight slices/condition/culture). The quantification of Western blot bands (Fig. 4) was performed using MetaMorph image analysis software (Universal Imaging Corporation, Downingtown, PA, USA). Bands that corresponded to Elmo1 and α-tubulin were manually traced, and their average area was automatically determined. The results are presented as the ratio of the average area of the Elmo1 band to the average area of the α-tubulin band.

### Timm’s Sulfate Silver Staining

To visualize aberrant axonal sprouting, Timm staining was used [37]. Slices still placed in culture inserts, were incubated for 10 min with 1 % sodium sulfide in 0.1 M phosphate buffer (PB), followed by the gentle addition of 0.3 % glutaraldehyde in PB [37] and incubation for another 5 min. The slices were then washed with PB and postfixed overnight at 4 °C with 70 % ethanol. The next day, the preparations were washed again with PB and incubated with development solution (50 % gum arabic solution, 0.5 M hydroquinone solution, 1.2 M citric acid, 0.8 M trisodium citrate, and 0.37 M silver lactate) for 30 min in the dark. After this step, the slices were washed with water, and Timm staining was fixed with 5 % thiosulfate for 12 min, followed by washing with water. The slices were then incubated with 70 % ethanol for 30 min, placed on gelatin-coated glass slides, dehydrated, cleared, and coverslipped. Timm’s staining was performed on two independent slice cultures (eight animals). Two to four slices per condition were analyzed.

### Statistical Analysis

The data were obtained from at least three batches of pooled slices or cultured cells. The results were analyzed using Prism software (GraphPad, La Jolla, CA, USA). For qRT-PCR analysis of *c-fos* expression and validation of microarray data, performed in slices and in vivo, we used Kruskal-Wallis test without following multiple comparisons tests due to low *n* of observations. For comparison of qRT-PCR and Western blot results obtained with use of cortical neurons, we used one-way ANOVA followed by Sidak’s multiple comparisons test. The Mann–Whitney test was used for the statistical analysis when two groups were compared. The statistical analysis of the microarrays was described in relevant section above.

## Results

### Neuronal Responses to Kainic Acid Are Mimicked in Hippocampal Organotypic Slices

A previous study indicated that KA-induced status epilepticus results in substantial changes in the transcriptome [38] and increases mTOR activity in adult animals [11]. Although mTOR regulates transcription [16], this specific aspect has not been previously studied in the context of proconvulsive drug actions. Thus, we investigated the effects of rapamycin on KA-induced changes in the transcriptome. We first established a simplified in vitro culture model that allowed the reduction of heterogeneity of animal responses to KA and excluded the necessity for prolonged and repetitive rapamycin injections. We used DIV5 organotypic hippocampal slices from newborn rats and tested their responses to KA and mTOR inhibition (Figs. 1 and 2). These preparations, similar to intact animals, responded to KA with robust and transient expression of *c-Fos*, an immediate early gene, at the mRNA and protein levels (Fig. 1b, c). *c-Fos* mRNA and protein expression reached a maximum at 2 h post-KA (Kruskal-Wallis test with no post hoc test due to low *n* of observations, *p* < 0.01 for 2 and 6 h) and returned to control levels at 24 h. We also tested our preparations for cell death, astrogliosis, and the sprouting of mossy fibers (i.e., long-term changes that are typically evoked by KA in vivo; [39]. Both TUNEL staining and the analysis of caspase-3 cleavage confirmed enhanced cell death in slices upon KA treatment (Figs. 1d and 2a). The immunofluorescent GFAP and MAP2 staining of slices confirmed that KA primarily affected survival of cells positive for MAP2 but not GFAP (Fig. 1d, e). GFAP immunostaining also revealed astrogliosis. Neuronal death that is induced by KA in the CA3 region of the hippocampus induces granule cells in the dentate gyrus to search for new synaptic targets, usually dendrites of other DG survivors. The formation of such aberrant connections, called aberrant sprouting, is a hallmark of epileptogenesis [40]. Timm’s sulfate silver staining revealed that KA that was added to organotypic hippocampal cultures induced mossy fiber sprouting 16 days post-KA. Consistent with previous in vivo results [11], rapamycin prevented this anatomical rearrangement (Fig. 1e). We next evaluated whether KA induces mTORC1 activity by monitoring the phosphorylation of ribosomal protein S6 (P-S6) at Ser 235/236. Similar to previously published results in intact animals [11], KA increased the level of P-S6 in a rapamycin-dependent manner (Fig. 2a, b). These results indicate that processes typically triggered by KA in vivo, like *c-Fos* expression and mTOR pathway induction, cell death, astrogliosis, and axonal sprouting can be also induced in organotypic hippocampal slices in response to KA.

### Gene Expression Profiling of Kainic Acid- and Rapamycin-Induced Transcriptional Changes

**Fig. 3:**
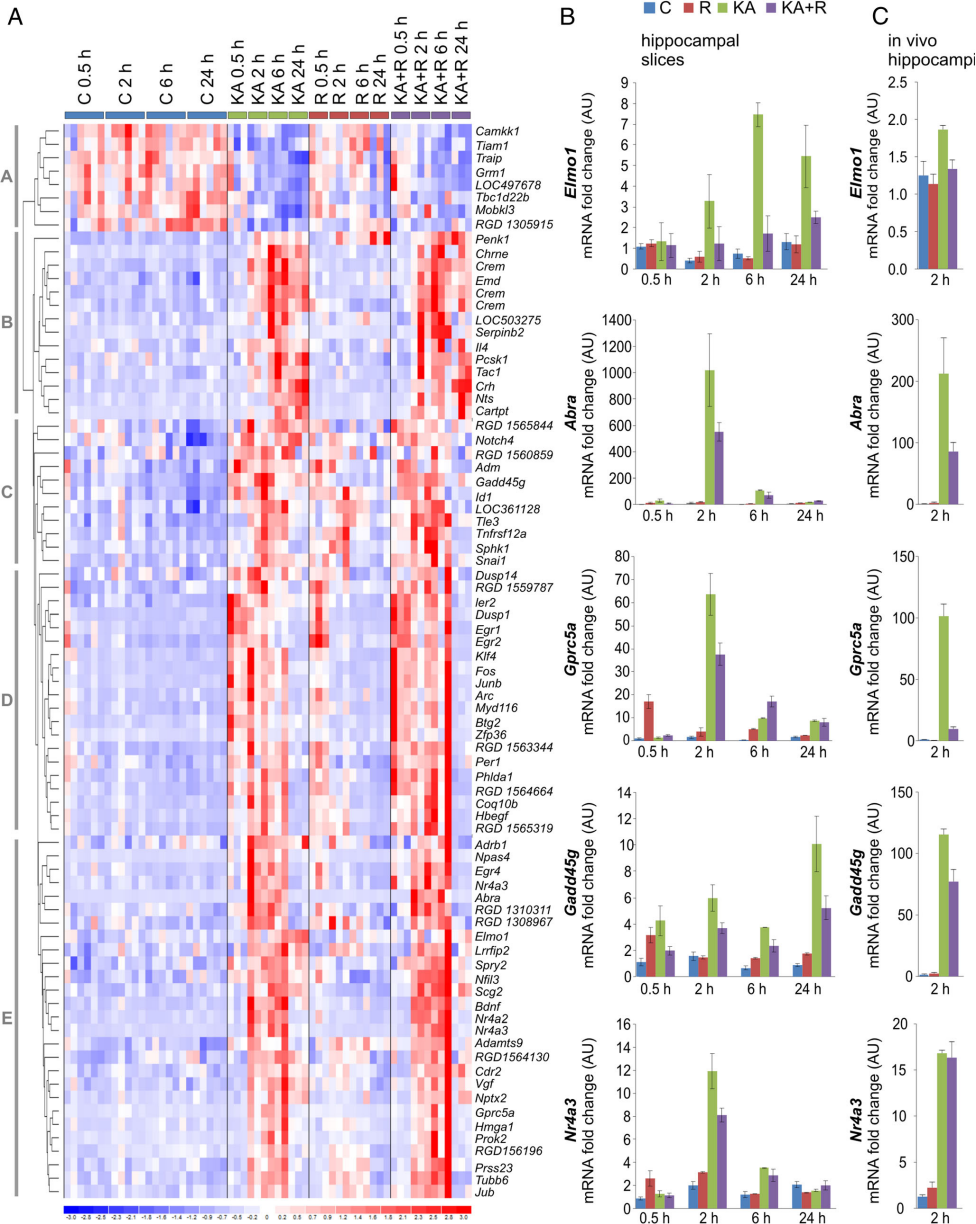
Hierarchical clustering of transcriptional alterations in response to KA in rat hippocampal slices and validation of selected gene expression changes. **a** Microarray results are shown as a heat map and include rat genes with genome-wide significance (two-way ANOVA of KA effects; FDR < 1 %). *Colored rectangles* represent transcript abundance of the genes labeled on the right. Gene expression was measured 0.5, 2, 6, and 24 h after KA, rapamycin (R), or KA+R treatments. The experimental groups are indicated above the heat map. The intensity of the color is proportional to the standardized values (between −3 and 3) from each microarray as indicated on the bar below the map image. Clustering was performed using Euclidean distance according to the scale on the left. KA-regulated genes were segregated into five time-dependent gene clusters (*A*–*E*). **b** Results of qRT-PCR-based analysis of indicated gene expression in organotypic hippocampal slices treated as indicated. The data are presented as mRNA fold changes relative to the control ± standard error (number of cultures [*N*] = 3; number of slices per culture: C, *n* = 16; R, *n* = 8; KA, *n* = 8; KA+R, *n* = 8. **c** Results of qRT-PCR-based analysis of indicated gene expression in hippocampi of rats treated as described in [11]. The data are presented as mRNA fold changes relative to the control ± standard error (number of animals: C, *n* = 3; R, *n* = 5; KA, *n* = 3; KA+R, *n* = 5)

Genomic research strategies have recently transitioned from the search for unknown genes to the identification and evaluation of coordinated gene networks and transcriptional signatures. Thus, to identify the potential contribution of mTOR to KA-evoked changes in the transcriptome, we used Illumina whole-genome rat microarrays to analyze the time course of gene expression alterations following KA administration in the presence or absence of rapamycin. To analyze the dynamics of early, intermediate, and relatively late changes in mRNA abundance, the analysis was performed 0.5, 2, 6, and 24 h after drug administration (Fig. 1a). We identified 81 KA-responsive genes (two-way ANOVA, FDR < 1 %), which were divided into five major clusters (A–E; Fig. 3a, Supplementary Table 1, Online Resource 1). The clusters revealed diverse KA-induced and time-dependent patterns of upregulation (clusters B–E) and downregulation (cluster A) of gene expression. Assuming the coordinated transcriptional regulation of identified genes, we searched for the overrepresentation of transcription factor binding sites. We found the overrepresentation of transcription factor binding sites, for such transcription factors as SRF (e.g., *Egr1*, *Egr2*, and *Npas4*) and CREB1 (e.g., *Ier2*, *Dusp1*, and *Fos*) in the promoter regions of the regulated genes (TFBS fold > 4; *p* < 0.05). As we expected, expression of the majority of these genes, present mostly in cluster D, was previously reported as dependent on neuronal activity. We also identified few genes regulated by rapamycin alone, including *Sphk1*, *Penk1*, and *LOC304131* (two-way ANOVA, FDR < 1 %; Supplementary Table 2 [Online Resource 2] and Supplementary Fig. 1 [Online Resource 3]). Rapamycin induced alterations in the KA-induced expression of genes from cluster C (0.5 h time point according to scheme of experiment), cluster B (0.5 h time point), and cluster E (2 h time point) (Supplementary Table 1, Online Resource 1). Specifically, these effects, although weak, were visible as the enhanced induction of several genes from cluster B at the 2 h time point and inhibition of the expression of genes from clusters C and E at 2, 6, or 24 h (Supplementary Table 1, Online Resource 1 and Supplementary Fig. 2, Online Resource 3). Based on this evidence, we concluded that mTORC1 inhibition has discrete rather than global impact on KA-evoked transcriptome changes.

### Effects of mTORC1 Inhibition on Kainic Acid-Induced Expression of Selected Genes

Although global analysis of the microarray data did not point to a group of genes with common characteristics that are regulated by mTORC1 in response to KA, of the list of genes that were differentially expressed between the KA and rapamycin+KA groups, we selected candidate genes, expression of which was downregulated by rapamycin (Supplementary Fig. 2, Online Resource 3), for further validation. We validated microarray results by qRT-PCR using total RNA that was isolated from organotypic hippocampal slices (mRNA aliquots were collected in parallel for both analyses). Detailed inspection of the microarray results revealed several genes, the levels of which differed between the KA and rapamycin+KA groups (e.g., *Tubb6*, *Bdnf*, *Elmo1*, *Lrrfip2*, *Npas4*, *Nr4a3*, *Abra*, *Cdr2*, *Vgf*, *Gprc5a*, *Nfil3*, *Scg2*, and *Egr4* [all from cluster E] and *Gadd45g* [cluster C]; Fig. 3a; Supplementary Table 1, Online Resource 1). The expression patterns of the majority of the analyzed genes were consistent with those that were revealed by the microarray analysis (Fig. 3b, Supplementary Fig. 3 [Online Resource 3]). We observed the impact of rapamycin on the effects of KA for *Elmo1*, *Abra*, *Gprc5a*, *Nr4a3*, *Vgf*, *Tubb6*, *Gadd45g*, and *Npas4*. In many cases, qRT-PCR is more sensitive than microarrays. Therefore, the effects of rapamycin were detected at additional time points (Fig. 3b, Supplementary Fig. 3A [Online Resource 3]). In the case of *Elmo1*, *Abra*, *Gprc5a*, *Nr4a3*, *Vgf*, *Tubb6*, and *Gadd45g*, we noted an impact of rapamycin on their KA-induced expression also at earlier time points. To further validate our observations, we analyzed by qRT-PCR expression of selected genes in hippocampi of rats 2 h post-KA. As shown in Fig. 3c and Supplementary Fig. 3B (Online Resource 3), expression of *Elmo1*, *Abra*, *Gprc5a*, *Nr4a3*, *Vgf*, *Npas4*, *Tubb6*, and *Gadd45g* substantially increased compared to control rats. At the same time, long-term rapamycin pretreatment (4 weeks; see [11] for detailed description of experiment and validation of rapamycin effectiveness as mRNA was obtained in course of experiments described there) considerably decreased KA-induced increases of *Elmo1*, *Abra*, *Gprc5a*, *Vgf*, and *Gadd45g* mRNA levels. Thus, we concluded that KA-induced mTORC1-dependent changes in the selected gene expression, identified by microarray approach, indeed occur, both in hippocampal slices cultured in vitro as well as in the brain in vivo.

### Long-Lasting Increase in Elmo1 Expression Affects Neuronal Morphology

**Fig. 4:**
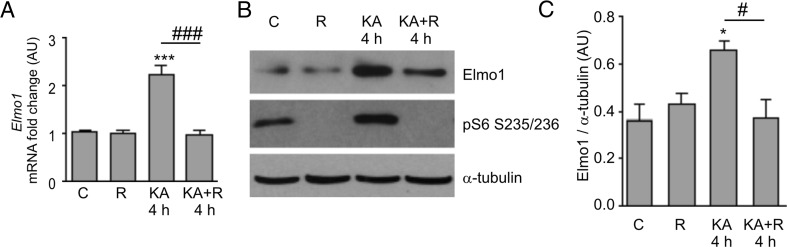
Kainic acid-induced *Elmo1* expression is mTOR-dependent in cultured cortical neurons. **a** Results of qRT-PCR-based analysis of *Elmo1* expression in cortical neurons cultured in vitro for 5 days and treated as indicated (*C* control, *R* rapamycin, *KA* kainic acid, *KA*+*R* KA+rapamycin). The data are presented as mRNA fold changes relative to the control ± standard error (number of cultures *N* = 5). Significant differences in transcript abundance between the treatments and controls are indicated by *asterisks* (***p < 0.001). Significant differences in transcript abundance between KA and KA+sR are indicated by hash signs (^###^ *p* < 0.001; one-way ANOVA followed by Sidak’s multiple comparisons test). **b** Representative result of Western blot analysis of Elmo1 expression in cortical neurons cultured in vitro for 14 days and treated as indicated. **c** Quantitative analysis of Western blot of Elmo1 expression levels in cortical neurons cultured in vitro for 14 days and treated as indicated (number of cultures *N* = 5). α-Tubulin levels were used for normalization. Significant differences in protein abundance between the treatments and controls are indicated by *asterisks* (*p < 0.05). Significant differences in protein abundance between KA and KA+R are indicated by hash signs (^#^ *p* < 0.05; one-way ANOVA followed by Sidak’s multiple comparisons test)

We next focused on *Elmo1* (cluster E) because its upregulation by KA was long-lasting and efficiently blocked by rapamycin at 6 and 24 h. What is more, Elmo1 was described to regulate actin cytoskeleton during neuronal development [26, 41]. To verify that KA-triggered changes in *Elmo1* expression translate into alterations at the protein level, we produced a new anti-Elmo1 antibody because none of those that are commercially available met our specificity criteria (e.g., all tested antibodies recognized several bands, none of which was affected by RNA interference; not shown). This newly generated antibody, which targets the C-terminus of Elmo1, recognizes overexpressed and endogenous proteins of proper size in cultured cortical neurons that are electroporated with β-actin-Elmo1-GFP (Supplementary Fig. 4, Online Resource 3). Moreover, the signal that corresponded to the endogenous signal weakened upon the transfection of neurons with a plasmid that encoded shRNA against *Elmo1* mRNA (Supplementary Fig. 4, Online Resource 3). Using this antibody, we sought to confirm the increase in Elmo1 protein in response to KA in our slice preparations. However, because of the low amount of protein, we did not obtain statistically significant confirmation. Therefore, to ensure a sufficient amount of material for the biochemical analysis, we switched to in vitro cultured cortical neurons. We first confirmed that KA treatment in these cells increased *Elmo1* mRNA levels in a rapamycin-dependent manner. As shown in Fig. 4a, *Elmo1* expression increased 4 h post-KA and rapamycin significantly prevented the effects of KA on *Elmo1* mRNA levels. Similar results were found for Elmo1 protein levels (Fig. 4b, c). Decrease of P-S6 levels additionally confirmed that rapamycin efficiently silenced mTOR signaling at the tested time points (Fig. 4b).

**Fig. 5:**
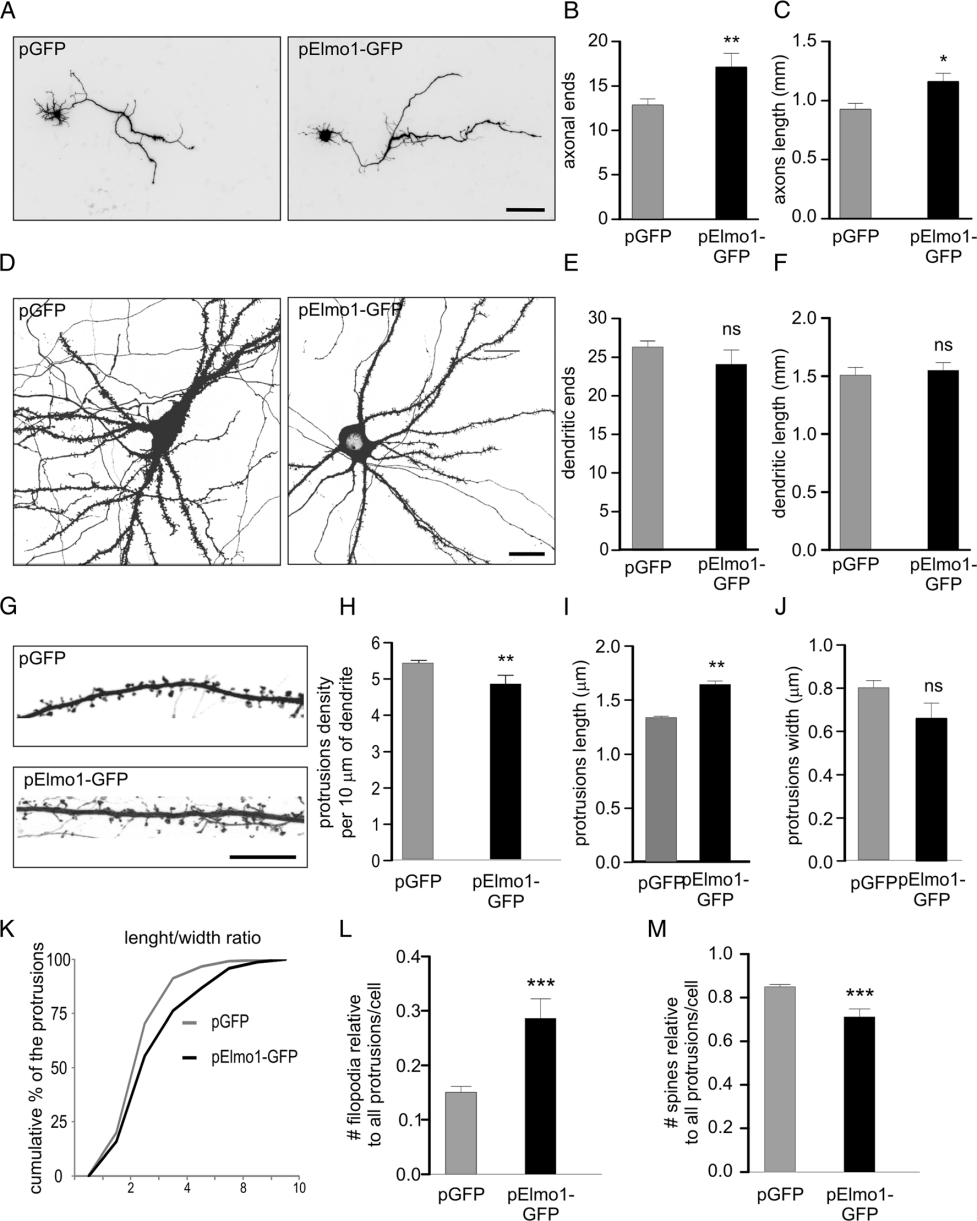
Elmo1 overexpression in hippocampal neurons affects axonal growth and dendritic spine morphology and density. **a** Representative confocal images of hippocampal neurons transfected on DIV1 with a plasmid that encoded GFP or Elmo1-GFP and fixed 3 days later. β-Actin-tdTomato was cotransfected to highlight transfected cell morphology. **b** Number of axonal ends and **c** axonal length of cells transfected as in **a**. *p < 0.05, **p < 0.01 (Mann–Whitney test). Cell images were obtained from three independent culture batches. Number of cells (*n*): GFP, *n* = 70; Elmo1-GFP, *n* = 58. *Scale bar* = 100 μm. **d** Representative confocal images of hippocampal neurons transfected on DIV8 with a plasmid that encoded GFP or Elmo1-GFP and fixed 3 days later. β-Actin-tdTomato was cotransfected to highlight transfected cell morphology. **e** Number of dendritic ends and **f** total dendrite length of cells transfected as in **d**. *ns* nonsignificant (Mann–Whitney test). Cell images were obtained from three independent culture batches. Number of cells (*n*): GFP, *n* = 58; Elmo1-GFP, *n* = 55. *Scale bar* = 20 μm. **g** Representative confocal images of dendrite segments of hippocampal neurons transfected on DIV18 with a plasmid that encoded GFP or Elmo1-GFP and fixed 3 days later. β-Actin-tdTomato was cotransfected to highlight transfected cell morphology. **h** Average protrusion density, **i** protrusion width, and **j** protrusion length per cell of cells transfected as in **g. k** Cumulative distribution of protrusion length to width ratio of neurons transfected as in **g. l**, **m** Contribution of spine and filopodia to the cell protrusion population of transfected cells. *ns* nonsignificant. **p < 0.01 (Mann–Whitney test). Number of independent cultures *N* = 2. Number of cells (*n*): GFP, *n* = 39; Elmo1-GFP, *n* = 36. *Scale bar* = 10 μm

Elmo1 is known to regulate F-actin dynamics, which underlies physiological and pathological rearrangements of neuronal morphology during development [26, 41]. Thus, we evaluated effects of the prolonged elevated expression of Elmo1 on the morphology of hippocampal neurons cultured in vitro at different developmental stages. Specifically, we morphometrically analyzed axonal and dendritic arbors of developing neurons and dendritic protrusions (spines and filopodia) of mature ones. Changes in axonal arborization and the number and shape of dendritic protrusions were previously reported in aberrant neuronal plasticity that accompanies epileptogenesis. We cotransfected cultured neurons for 3 days with β-actin-Elmo1-GFP and β-actin-tdTomato (to highlight the morphology of transfected neurons) and analyzed their morphology. We used DIV1, −7 developing, and −18 mature neurons for the analysis of axons, dendrites, and dendritic protrusions, respectively. As shown in Fig. 5a–c, axon length and number of axonal ends significantly increased upon Elmo1-GFP overexpression. In contrast, increases in the levels of Elmo1 did not affect either number of dendritic tips or total dendritic length (Fig. 5d–f). In case of dendritic protrusions the morphometric analysis revealed that cells that were transfected with β-actin-Elmo1-GFP had a lower density of dendritic protrusions compared with controls, which were also in average significantly longer (Fig. 5g–i). We also observed, tendency for dendritic protrusions width decrease in Elmo1 overexpressing neurons, which did not however reach statistical significance (Fig. 5j). Analysis of length to width ratio [32] indeed confirmed substantial shift of protrusion morphology toward filopodia-like one (Fig. 5k). Indeed, close inspection of specific frequencies of filopodia and spines in Elmo1 overexpressing cells revealed significant increase and decrease in filopodia and spine numbers, respectively (Fig. 5l, m). This suggests that Elmo1 overexpression in mature neurons, as oppose to developing ones [26] leads to spine destabilization and elimination.

## Discussion

mTOR inhibition has been considered for antiepileptogenic therapy [42, 43]. Epileptogenesis has been established to be associated with long-term changes in gene expression. We used a simplified in vitro culture system to investigate whether rapamycin affects transcriptome changes that are evoked by KA. We found that processes typically triggered by KA in vivo, like *c-Fos* expression and mTOR pathway induction, cell death, astrogliosis, and axonal sprouting were also induced in organotypic hippocampal slices in response to KA. The microarray analysis revealed changes in the transcription profiles of several genes in response to KA. Rapamycin affected only some of those changes, for example by preventing *Elmo1*, *Abra*, *Gprc5a*, *Nr4a3*, *Vgf*, *Tubb6*, *Gadd45g*, and *Npas4* expression upregulation, suggesting that mTORC1 is not massively affecting KA-evoked transcriptome changes. We further focused on Elmo1, a modulator of actin dynamics and found that Elmo1 overexpression in hippocampal neurons increased axonal arborization and decreased dendritic spine density.

### Effects of Kainic Acid on Organotypic Cultures in the Context of In Vivo Data

In this work, we described a simplified in vitro model to study molecular changes that are induced by KA. Importantly, we showed that the essential hallmarks of in vivo epileptogenesis (e.g., the induction of *c-Fos* expression, astrogliosis, neuronal death, and aberrant axonal sprouting) were reproduced in organotypic hippocampal slices in response to KA. One issue that arose was the extent to which our gene profiling results are consistent with data from in vivo models. Multiple gene profiling studies have been conducted using animal models of epilepsy [44]. Differentially expressed genes have been reported in status epilepticus or poststatus epilepticus rodent models, e.g. application of KA [45, 46] and pilocarpine [47, 48], or electrical stimulation [49, 50]. Notably, despite the large amount of data that have already accumulated, a comparative meta-analysis is difficult to perform because of the different experimental designs and microarray platforms that have been used in previous studies. Our data overlap to a largest extent with those of Kuzniewska et al. [46], who performed gene profiling in response to intrahippocampus-administered KA. Among the overlapping genes were *Elmo1*, *Gadd45g*, *Npas4*, *Egr4*, *Gprc5a*, *Scg2*, *Nr4a3*, *Egr1*, and *Zfp36*. Also, other genes identified by us were previously reported in animal models of epilepsy, including *c-Fos* and *c-Jun* [51], *Bdnf* [52], *Dusp1* [53], *Arc* [54], *Penk1* [55–57], *Egr2* [58], *Crem* [59], and *Id1* [60, 61]. Thus, we conclude that our simplified model recapitulates major molecular and cellular changes that are observed in in vivo models of epilepsy. Consistently, several genes, expression of which was induced by KA in rapamycin-dependent manner in organotypic hippocampal cultures were controlled in similar way in vivo (Fig. 3c).

### Effects of Rapamycin on Kainic Acid-Induced Gene Expression

Several recent reports suggest that the mTOR pathway may be central to epileptogenesis and therefore could be a convenient therapeutic target [14, 62]. Rapamycin has antiepileptogenic effects (e.g. prevents aberrant mossy fiber sprouting, appearance of recurrent seizures) in models of epilepsy in animals [12–15]. In the present study, we focused on the influence of rapamycin on mRNA expression profiles under basal conditions and in response to KA. Our major observation was that rapamycin influences the expression of a small subset of genes induced by KA, rather than globally counteracts changes induced in transcriptome by KA. This is consistent with multiple actions of rapamycin including decrease in protein synthesis and induction of autophagy, which both may contribute to epileptogenesis prevention [63, 64]. Still it raises a question, what is the molecular mechanism that underlies the effects of rapamycin on KA-driven gene expression and provides observed selectivity of rapamycin effects. Because gene profiling only reveals the amount of a transcript, one should consider the role of mTOR in either the regulation of transcription or mRNA stabilization. In nonneuronal cells, mTOR is known to regulate activity, cellular localization, and the level of several transcription factors [16], e.g., hypoxia-inducible factor 1α (HIF-1α), increased expression of which was reported in animal models of epilepsy [65, 66]. However, in our analysis, we did not identify TFBS for HIF-1α in promoters of KA-upregulated genes that were affected by rapamycin. However, several rapamycin-modulated genes (e.g., *Elmo1*, *Gadd45g*, and *Npas4*) were also identified in a recent transcriptome study by Kuzniewska et al. [46], as controlled by SRF in response to KA. Additionally, chronic rapamycin treatment in rats and SRF knockout in the mouse forebrain increased the susceptibility to seizures and their severity [11, 46]. One issue is the way in which mTOR influences SRF transcriptional activity. Although no potential mechanism has been described to date, the present results identified *Abra* (STARS) as a rapamycin-modulated gene. The *Abra* product activates SRF-dependent transcription, inducing the nuclear translocation of megakaryoblastic leukemia 1 (MKL1) or MKL2 [67]. Thus, rapamycin could theoretically specifically downregulate SRF-dependent transcription to regulate the cellular localization of MKLs.

An alternative explanation for the contribution of mTOR to KA-induced gene expression is its involvement in transcript stabilization. mTOR participates in this process in nonneuronal cells. For example, rapamycin decreases the steady-state level of collagen (*COL1A1* and *COL1A2*) mRNA in fibroblasts and destabilizes inducible nitric oxide synthase mRNA in astrocytes and transient receptor potential melastatin 6 in renal tubular epithelial cells [68–70]. Whether mTOR stabilizes transcripts that are induced by status epilepticus remains to be investigated.

### Potential Contribution to Kainic Acid-Related Neuropathology at the Cellular Level

Our data suggest that mTOR inhibition modulates the KA-induced expression of a subset of genes, but unknown is whether it has a potential impact on epileptogenesis. Knowing their cellular functions, we hypothesize that rapamycin could modulate cell death and cytoskeleton rearrangements, which both contribute to epileptogenesis. For example, Gadd45g plays a role in neuronal apoptosis during KA-dependent status epilepticus [71]. Moreover, Abra [72, 73], Elmo1 [74, 75], and Nr4a3 [76] were previously implicated in apoptosis. In our model, however, we could not prove antiapoptotic effects of rapamycin (see Fig. 2 for lack of an effect of rapamycin on KA-induced cleavage of caspase-3). Therefore, one possibility is that rapamycin, although it affects the expression of potentially proapoptotic genes, is unable to prevent the first wave of excitotoxic cell death upon KA application. Among our candidates, the products of three genes (*Tubb6*, *Abra*, and *Elmo1*) are known to modulate the cytoskeleton. *Tubb6* encodes β-tubulin, and dynamic microtubules contribute to axonal growth and spine shape changes [30, 77, 78]. Nonetheless, the particular functions of this β-tubulin isoform in the nervous system have not been described but its deletion may contribute to microcephaly (Jawad syndrome; [79]). Abra increases the actin polymerization of muscle cells [67], but its role in neurons has not been described. In theory, its lowered expression in response to rapamycin could prevent KA-induced neuronal changes in dendritic spine morphology or mossy fiber sprouting. Elmo1 is also a regulator of RhoA and Rac1. Via these small GTPases, it regulates actin dynamics. The potential contribution of Elmo1 to KA-induced changes is discussed below.

### Potential Contribution of mTOR-Dependent Elmo1 Regulation to Pathology

In the present study, we identified *Elmo1* as a gene whose expression is activated by KA and is modulated by rapamycin. Using cortical neuron cultures in vitro, we also demonstrated that changes in the amount of *Elmo1* mRNA correlate well with changes in Elmo1 protein levels. Elmo1 is a regulator of RhoA and Rac1 and via these small GTPases Elmo1 regulates actin cytoskeleton. Elmo1 was shown to participate in neuronal apoptosis and neurite outgrowth [80]. Thus, in our in vitro model, the KA-induced expression of *Elmo1* would be associated with reorganization of the cytoskeleton. Consequently, the downregulation of *Elmo1* expression could explain the effects of rapamycin on KA-induced neuronal morphological changes, such as axon sprouting and spine shape rearrangements, which depend on the actin cytoskeleton. In the present study, we investigated effects of increased Elmo1 expression on morphology of developing and mature hippocampal neurons cultured in vitro. In case of developing cells, we reported that overexpression of Elmo1 induces axonal growth and arborization. This result corroborates potential role of Elmo1 in epilepsy-related sprouting but does not prove it because epilepsy-related sprouting occurs in mature cells. Unfortunately, for technical reasons, it was impossible to undoubtedly trace axons of mature neurons in our type of culture. Yet, our observation brings new information concerning Elmo1 and suggests that cellular availability of this protein may limit axonal growth during development. This result is somewhat contradictory with work of Franke et al. [41], who showed that RhoG/Elmo1/Dock180/Rac1 pathway restricts axonal branching of embryonic hippocampal neurons. Yet, Franke et al. [41] used mutant Elmo1, which can not bind Dock180 (Elmo1-D625) but did not study wild type Elmo1. Thus, one can speculate that Elmo1 possesses progrowth activity which is mitigated by Dock180. Alternatively, it is possible that Elmo1 acts also outside its canonical pathway during axonal growth, but in such case, the role of Rac1 and actin cytoskeleton cannot be readily claimed.

Another novel finding reported herein is that increased Elmo1 level in mature hippocampal neurons resulted in reduction of dendritic spine number as well as induced their shape change to filopodia-like one. Thus, increased Elmo1 expression in mature neurons, a model relevant to developmental stage for which we performed gene profiling experiments, seems to drive spine elimination. Notably, decreases in spine number have been reported in mouse brain 24 h post-KA [81]. It would be of interest to check if rapamycin prevents spine elimination induced by KA. In addition, our result suggests that Elmo1 plays different role in developing and mature neurons with regard to dendritic spines since Kim et al. [26], overexpressed Elmo1 in developing neurons at the advent of spinogenesis (DIV6) and reported slight increase in number of dendritic spines by DIV18.

In conclusion, data presented herein show that increased activity of mTORC1 in response to kainic acid is not affecting gene expression globally. However, our findings suggest that rapamycin, in addition to previously described activities, may indeed affect development of epilepsy, by modulating expression of specific subset of genes, including *Elmo1* and point to a potential role for Elmo1 in morphological changes that accompany epileptogenesis.

## Electronic supplementary material

Below is the link to the electronic supplementary material.

Online resource 1 (XLS 119 kb)

Online resource 2 (XLS 25 kb)

Online resource 3 (PDF 407 kb)
