# Supplementary material for: Kainic Acid Induces mTORC1-Dependent Expression of Elmo1 in Hippocampal Neurons
Source: Mol Neurobiol. 2016 Mar 19;54(4):2562–78. doi: 10.1007/s12035-016-9821-6 (PMC5390005; doi:10.1007/s12035-016-9821-6)
Supplement: Supplementary file 3 — (PDF 407 kb) [file 12035_2016_9821_MOESM3_ESM.pdf]

Blazejczyk et al. - Online Resource 3. Supplementary Figures

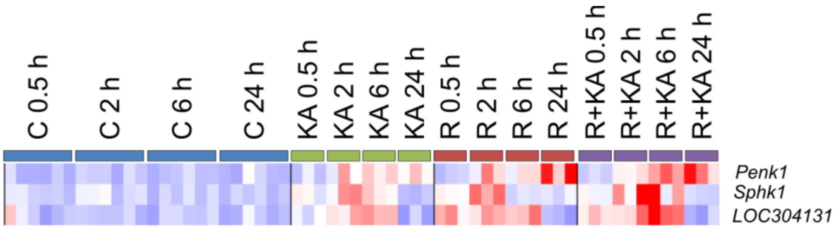

Supplementary Fig. 1. Hierarchical clustering of transcriptional alterations in response to rapamycin in rat hippocampal slices. Microarray results are shown as a heat map and include rat genes with genome-wide significance (two-way ANOVA of R effects; FDR < 1%). Colored rectangles represent transcript abundance of the genes labeled on the right. Gene expression was measured 0.5, 2, 6, and 24 h after KA, rapamycin (R), or R+KA treatments. The experimental groups are indicated above the heat map. The intensity of the color is proportional to the standardized values (between -3 and 3) from each microarray as indicated on the bar below the map image.

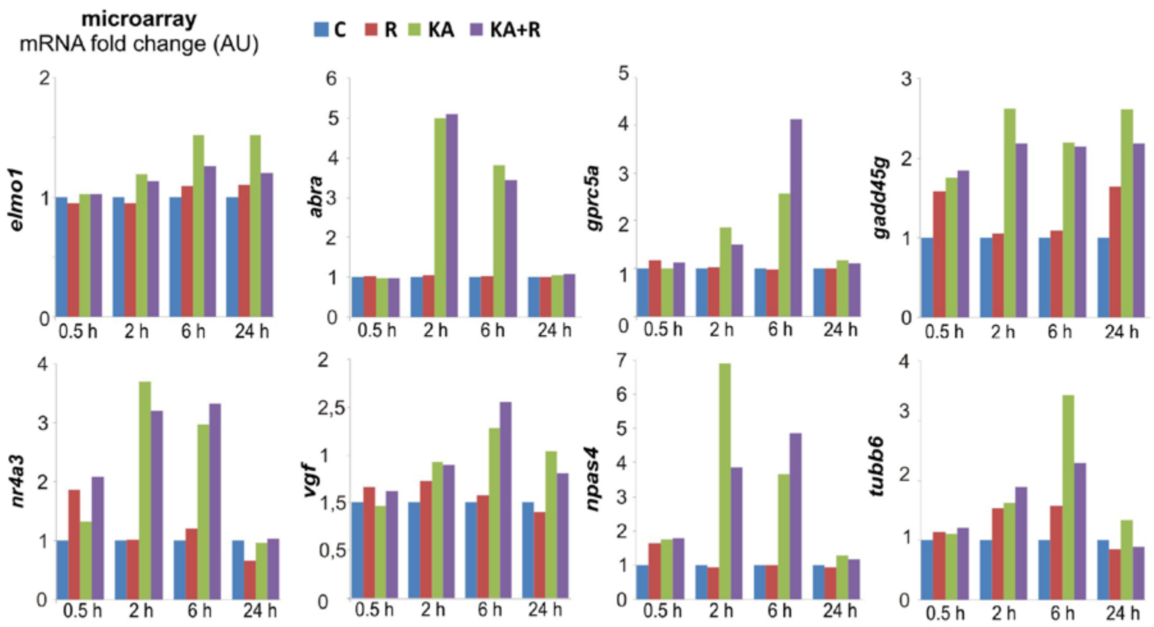

Supplementary Fig. 3. Bar graphs representation of microarray data for selected genes KA-induced expression of which was partly prevented by rapamycin at 2, 6 or 24 h. C - control, R - rapamycin, KA - kainic acid; KA+R - kainic acid + rapamycin.

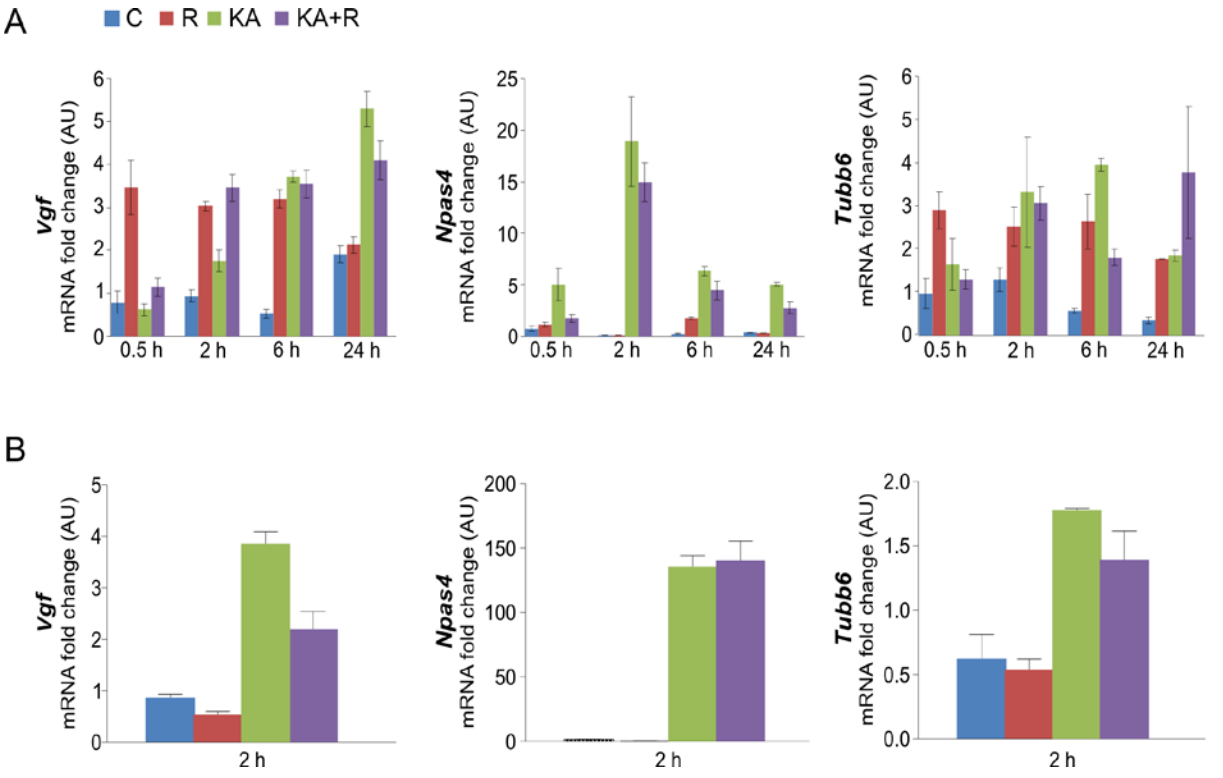

Supplementary Fig. 3. Validation of additional genes selected from microarray data. (A) The results of the qRT-PCR-based analysis of indicated gene expression in organotypic hippocampal slices treated as indicated (C - control, R - rapamycin, KA - kainic acid; KA+R - kainic acid + rapamycin) are shown. The data are presented as mRNA fold changes relative to the control ± standard error (three biological repeats). (B) Results of qRT-PCR-based analysis of indicated gene expression in hippocampi of rats treated as described in [1]. The data are presented as mRNA fold changes relative to the control ± standard error (number of animals: C, n = 3; R, n = 5; KA, n = 3; KA+R, n = 5).

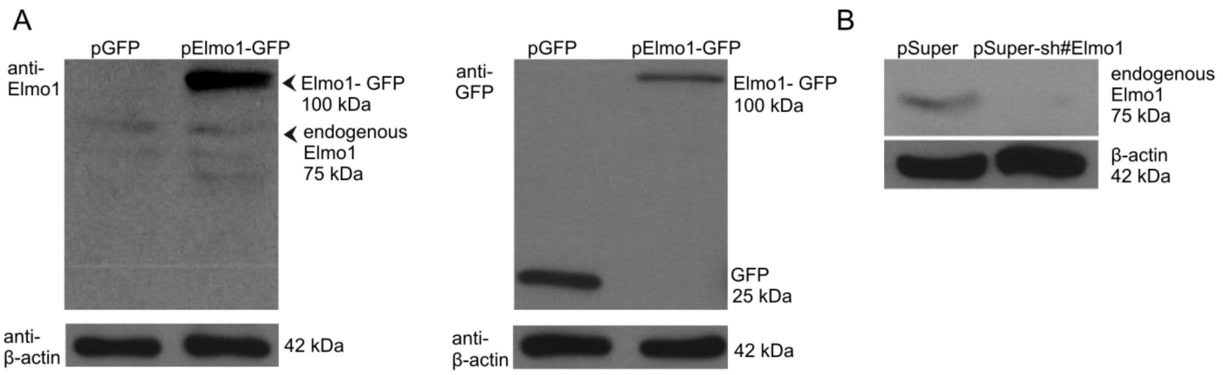

Supplementary Fig. 4. Analysis of Elmo1 antibody specificity. (A) Analysis of effectiveness and specificity of Elmo1 antibody in protein extracts obtained from primary cortical neurons that overexpressed β-actin-Elmo1-GFP (pElmo1-GFP) or β-actin-GFP (pGFP). GFP immunodetection was performed as a control for the presence of overexpressed Elmo1-GFP. (B) Analysis of specificity of Elmo1 antibody in protein extracts obtained from primary cortical neurons nucleofected with empty pSuper vector or pSuper-sh#Elmo1. β-actin is shown as a loading control.

Supplementary Figures References

1. Macias M, Blazejczyk M, Kazmierska P, Caban B, Skalecka A, Tarkowski B, Rodo A, Konopacki J, Jaworski J: Spatiotemporal characterization of mTOR kinase activity following kainic acid induced status epilepticus and analysis of rat brain response to chronic rapamycin treatment. *PLoS One* 2013, 8:e64455.
